# Synchrotron Radiation‐Based Tomography of an Entire Mouse Brain with Sub‐Micron Voxels: Augmenting Interactive Brain Atlases with Terabyte Data

**DOI:** 10.1002/advs.202416879

**Published:** 2025-04-29

**Authors:** Mattia Humbel, Christine Tanner, Marta Girona Alarcón, Georg Schulz, Timm Weitkamp, Mario Scheel, Vartan Kurtcuoglu, Bert Müller, Griffin Rodgers

**Affiliations:** ^1^ Biomaterials Science Center Department of Biomedical Engineering University of Basel Hegenheimermattweg 167B/C Allschwil 4123 Switzerland; ^2^ Core Facility Micro‐ and Nanotomography Department of Biomedical Engineering University of Basel Hegenheimermattweg 167B/C Allschwil 4123 Switzerland; ^3^ The Interface Group Institute of Physiology University of Zurich Winterthurerstrasse 190 Zurich 8057 Switzerland; ^4^ Synchrotron SOLEIL, L'Orme des Merisiers Saint‐Aubin 91190 France; ^5^ Biomaterials Science Center Department of Clinical Research University Hospital Basel Spitalstrasse 8/12 Basel 4031 Switzerland

**Keywords:** big data, extended field‐of‐view, multi‐resolution open data, neuroimaging, registration, X‐ray histology

## Abstract

Synchrotron radiation‐based X‐ray microtomography is uniquely suited for post‐mortem 3D visualization of organs such as the mouse brain. Tomographic imaging of the entire mouse brain with isotropic cellular resolution requires an extended field‐of‐view and produces datasets of multiple terabytes in size. These data must be reconstructed, analyzed, and made accessible to domain experts who may have limited image processing knowledge. Extended‐field X‐ray microtomography is presented with 0.65μm voxel size covering an entire mouse brain. The 4495 projections from 8 × 8 offset acquisitions are stitched to reconstruct a volume of 15000^3^ voxels. The microtomography volume was non‐rigidly registered to the Allen Mouse Brain Common Coordinate Framework v3 based on a combination of image intensity and landmark pairs. The data were block‐wise transformed and stored in a public repository with a hierarchical format for navigation and overlay with anatomical annotations in online viewers such as Neuroglancer or siibra‐explorer. This study demonstrates X‐ray imaging and data processing for a full mouse brain, augmenting current atlases by improving resolution in the third dimension by an order of magnitude. The 3.3‐teravoxel dataset is publicly available and easily accessible for domain experts via browser‐based viewers.

## Graphical abstract

Extended‐field acquisition enables sub‐micron X‐ray imaging of a full mouse brain. The 3.3‐teravoxel volume is reconstructed, registered to a reference atlas, and stored in hierarchical format to augment currently available atlases and improve resolution in the third dimension by an order of magnitude. The data are publicly available and easily accessible via browser‐based viewers.

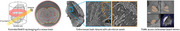

## 1 Introduction

Understanding the relationship between structure and function in the brain is a unique challenge and requires high‐resolution brain imaging.^[^ ^1^ ^]^ This is complicated by the fact that the length scales of structures of interest span many orders of magnitude, even for smaller mammalian species. The mouse brain has a width of about 1 cm, while individual cells are typically 1μmto10μm in size, and the width of synaptic connections can be below 100 nm.^[^ ^1^ ^]^ Three‐dimensional mapping of the brain's cytoarchitecture over these length scales requires complementary imaging modalities. X‐ray micro‐ and nanotomography are promising techniques for volumetric imaging of physically soft tissue, with hard X‐ray microtomography offering non‐destructive imaging at micrometer or sub‐micrometer resolution.^[^ ^2^, ^3^, ^4^, ^5^, ^6^, ^7^ ^]^ X‐ray nanoholotomography, where geometric magnification with a highly focused X‐ray beam is combined with multiple‐distance images to provide a quantitative phase map,^[^ ^8^ ^]^ allows for visualization of structures down to tens of nanometers in size.^[^ ^9^, ^10^ ^]^ While these techniques have been successfully employed for brain tissue, usually only sub‐volumes on the order of 10  mm^3^ are accessible, compared to a volume of 500  mm^3^ for the full mouse brain.^[^ ^11^ ^]^ Bridging this gap toward full‐brain microtomography at cellular resolution has become an active area of research in recent years.^[^ ^12^, ^13^, ^14^, ^15^, ^16^ ^]^

The field‐of‐view (FOV) offered by X‐ray microtomography detector systems is limited by the number of pixels and the desired pixel size. Taking the Hamamatsu Orca Flash 4.0 V2 detector in use at the anatomix beamline of Synchrotron soleil ^[^ ^17^, ^18^ ^]^ as an example, the camera has 2048 × 2048 pixels and a pixel size of 6.5μm. With a 10× magnifying objective, this gives a FOV of 1.3 mm × 1.3 mm. Thus, the FOV needs to be extended to cover the entire mouse brain. Extending the FOV in the vertical direction, i.e., parallel to the tomographic rotation axis, is possible by translation of the specimen along the rotation axis, either in a helical scan with simultaneous rotation and vertical translation, or by acquiring multiple scans at distinct height steps and stitching the reconstructed volumes together. Two approaches have been pursued for lateral extension of the FOV. One approach is to acquire single FOV tomograms and translate the sample on top of the rotation stage between scans to cover the sample's entire cross section. The volumes are then stitched after reconstruction.^[^ ^14^, ^19^ ^]^ An alternative scheme is to translate the rotation axis laterally with respect to the detector and acquire images over 360° for each rotation axis position, which can then be stitched to produce extended‐field projections.^[^ ^12^, ^20^ ^]^ The first approach has the advantage that it allows for standard reconstruction of each tomogram. It is also robust to some degree of sample deformations during acquisition when combined with non‐rigid stitching of reconstructed volumes.^[^ ^14^ ^]^ However, this acquisition approach requires correction of artifacts arising from reconstruction of truncated sinograms (also known as the *local tomography* problem).^[^ ^21^, ^22^, ^23^ ^]^ The second approach requires dedicated software for projection stitching and large‐volume reconstruction.^[^ ^12^, ^15^, ^20^ ^]^ The tilt angles of the rotation stage need to be aligned more precisely with respect to both the beam direction and the detector pixel rows than for standard microtomography scans. The second approach has, however, been shown to be more dose‐ and time‐efficient.^[^ ^13^ ^]^

The resulting dataset covering the volume of the entire mouse brain with 0.65μm voxel size will require 3 TB of storage space at 16‐bit precision. Sharing such a large image with the neuroscience community brings challenges in terms of data access, navigation, registration, and segmentation. To leverage existing brain atlas annotations and allow the community to better navigate the large dataset, the microtomography volume must be registered to an atlas. Yet the registration of large volumes often proves challenging, as it can lead to excessive runtimes or be constrained by memory limitations. To address these issues, we developed a distributed multi‐resolution approach, where the large volumes are divided hierarchically into regions to circumvent memory limitations. This approach was used to register a multi‐terabyte sized microtomography volume to the Allen Mouse Brain Common Coordinate Framework v3 (CCFv3).^[^ ^24^ ^]^

The effective sharing of research data within multidisciplinary communities requires easy access and interpretability. For imaging data, this ideally means online tools for visualizing, sharing, and annotating volumes instead of large repositories with only data download options. Recently, interactive visualization platforms have been developed for displaying volumes of mega‐ to petavoxel size, including siibra‐explorer ^[^ ^25^ ^]^ and Neuroglancer.^[^ ^26^ ^]^ siibra‐explorer is a browser‐based viewer for brain atlases that allows seamless querying of semantically and spatially anchored datasets thanks to tight integration with the Human Brain Project Knowledge Graph. Neuroglancer is a multi‐resolution viewer capable of displaying tera‐ to petavoxel‐sized datasets and their segmentations fast enough to be practical. The location and orientation of any view are encoded in the uniform resource locator (URL), which can be shared for easy collaboration. As open‐source software, Neuroglancer is highly extensible. Its usefulness for collaborative brain studies has been demonstrated, for example, for segmenting the full adult fly brain dataset at 4 × 4 × 40  nm^3^ resolution, resulting in 115 TB of image data.^[^ ^27^ ^]^ Given these promising properties, we converted the registered microtomography volume of an entire mouse brain to the efficient pre‐computed Neuroglancer format, which can also be read by siibra‐explorer, and then uploaded it to ebrains ^[^ ^28^ ^]^ for dissemination.

This work builds on the reconstruction pipeline for extended field‐of‐view tomographic reconstruction of entire organs with cellular resolution initially presented by Rodgers and co‐workers.^[^ ^29^ ^]^ Here, we report on i) the acquisition of a large microtomography volume of an entire mouse brain with a pixel size of 0.65μm and its ii) reconstruction, iii) registration to the Allen Mouse Brain CCFv3 atlas, iv) conversion to hierarchical Neuroglancer format for fast interactive visualization and access, and v) dissemination via the ebrains Data and Knowledge services. This enables the imaging of an entire mouse brain with three‐dimensional micrometer resolution and efficient sharing of the microtomography volume.

## 2 Results and Discussion

### 2.1 The Full Mouse Brain Dataset

**Figure 1:**
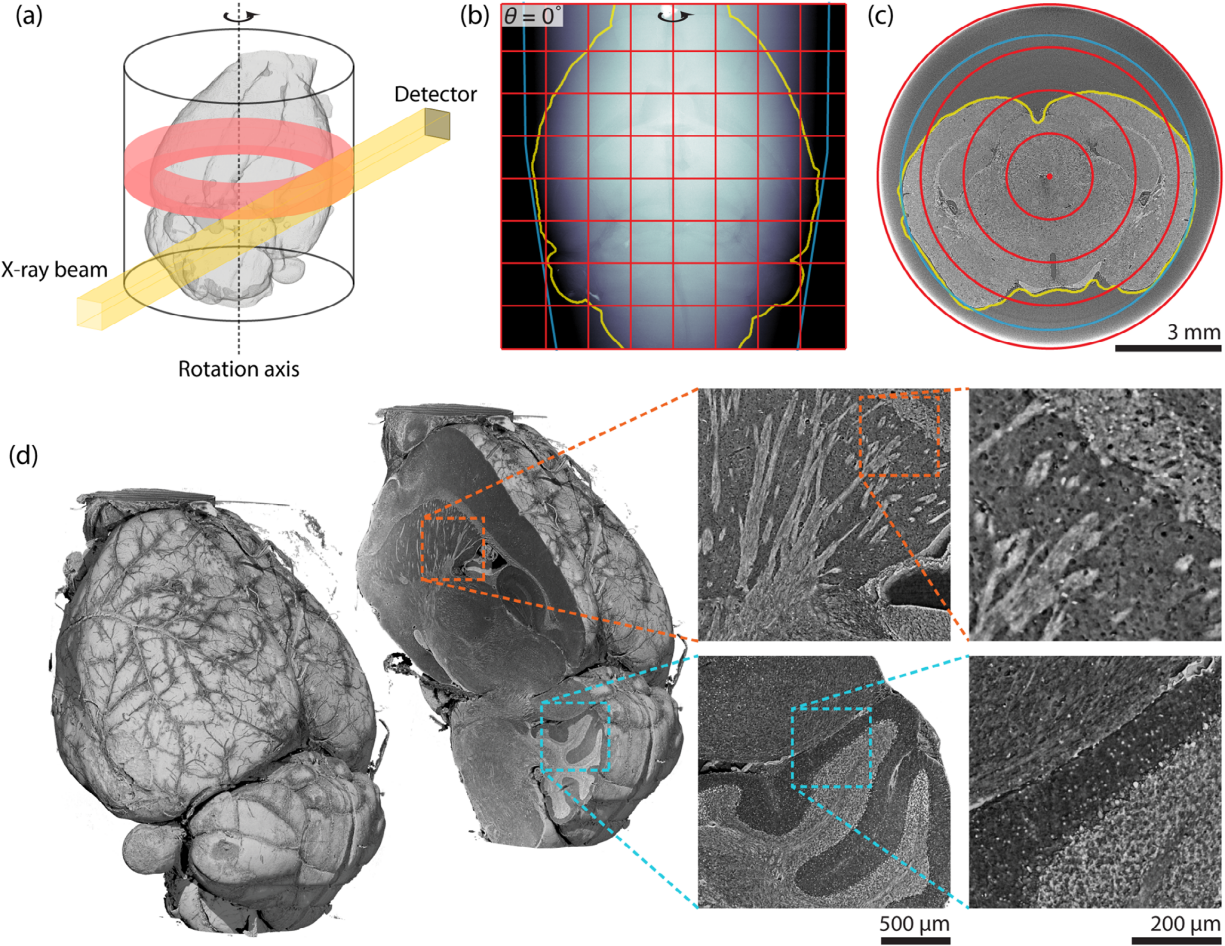
Large field‐of‐view (FOV) X‐ray microtomography of the entire mouse brain. To successively illuminate the entire cross‐sectional area, the rotation axis was displaced relative to the detector and the X‐ray beam (a). An exemplary stitched projection is shown at 0 rotation angle for the acquisition of the mouse brain with 8 × 8‐times extended FOVs (b). Red lines delineate single 2048 × 2048‐pixel detector FOVs that were combined with an overlap of 200 pixels to form a single 14982 × 14982‐pixel projection. A reconstructed slice is shown with corresponding stitching positions in red (c). Yellow lines indicate the outline of the mouse brain and blue lines indicate the inner wall of the Eppendorf container used as a sample holder (b, c). After reconstruction, the volumetric data can be viewed along any virtual slicing direction (d). Views from a sagittal slice are shown, alongside enlarged regions in the caudoputamen (orange) and cerebellum (cyan).

The full mouse brain, defined here to include the cerebellum but not the olfactory bulbs, was scanned with eight height steps, see **Figure** **1**b (caudal part of olfactory bulbs at top, brain stem at the bottom). The resulting stitched dataset contained 14982 × 14982 × 14784 voxels, or 3.3 teravoxels. At 16‐bit precision, this corresponds to a data size of 6.6 TB (where 1 TB = 10^12^ bytes). It should be noted that this reflects the entire reconstructed FOV of 911  mm^3^. Re‐orienting and cropping to the approximately 500  mm^3^ volume of the mouse brain would reduce data size.

Figure 1d (left) shows an overview rendering of the entire mouse brain data with 8 × 8 × 8 downsampling. Virtual sagittal sectioning as in Figure 1d (right) reveals macroscopic regions such as the isocortex, olfactory areas, hippocampal formation, cerebral nuclei, fiber tracts, and cerebellum. Renderings of the full‐resolution data in Figure 1d (right) provide in‐plane details similar to conventional histology, but with the benefit of 3D isotropic resolution. This allows for exploration of the volumetric data in any virtual slicing plane.

X‐ray microtomography belongs to a group of imaging techniques used for mapping brain structures that are three‐dimensionally organized across multiple length scales.^[^ ^1^ ^]^ MRI can provide in vivo or in situ neuroimaging of macroscopic brain structures, but it only reaches voxel sizes down to slightly below 1 mm. Magnetic resonance microscopy can extend resolution to tens of μm,^[^ ^30^ ^]^ though it lacks true cellular resolution. Conventional histology based on optical microscopy is the gold standard for evaluation of post‐mortem, sectioned brain tissue with sub‐cellular resolution. Out‐of‐plane spatial resolution is limited by section thickness, which is typically on the order of tens of μm. For example, the highest resolution histology‐based atlas of the entire human brain has 20μm out‐of‐plane resolution.^[^ ^31^ ^]^ Advanced methods combining two‐photon, light‐sheet, or confocal microscopy with sectioning via microtome or laser ablation^[^ ^32^, ^33^, ^34^ ^]^ have allowed for volumetric imaging of the entire mouse brain with isotropic 1μm voxels.^[^ ^35^, ^36^ ^]^ Light scattering can also be reduced by tissue clearing, allowing optical sectioning for volumetric imaging with high out‐of‐plane resolution.^[^ ^37^, ^38^, ^39^ ^]^ The disadvantages of these approaches include time‐consuming acquisition and specialized sample preparation. Hard X‐ray microtomography offers isotropic micrometer resolution for post‐mortem samples without clearing or physical sectioning of tissue. The combination of highly brilliant X‐rays from synchrotron radiation facilities or advanced laboratory sources with phase contrast techniques provides excellent contrast for neuroimaging.^[^ ^3^, ^4^, ^5^, ^6^, ^40^ ^]^ X‐ray microtomography is compatible with subsequent optical microscopy, and several studies have shown a satisfactory spatial agreement and comparable in‐plane resolution between the modalities.^[^ ^2^, ^40^ ^]^ Generally, MRI, histology, and microtomography are complementary techniques^[^ ^41^ ^]^ that can be combined to cover length scales from the entire brain to sub‐cellular details. Compared to histology, X‐ray microtomography offers approximately equal in‐plane and superior out‐of‐plane resolution, though histology has high specificity thanks to staining. Compared to MRI techniques, X‐ray microtomography has higher spatial resolution and is capable of cellular and sub‐cellular analysis, while MRI offers better in vivo and in situ neuroimaging possibilities. X‐ray nanotomography can provide post‐mortem neuroimaging with resolutions beyond the optical limit,^[^ ^9^ ^]^ albeit in smaller volumes compared to microtomography.

Imaging the in vivo mouse brain with microtomography at a comparable spatial resolution and contrast to the present post‐mortem data is currently impossible. Regions‐of‐interest in rat lungs were imaged with in vivo microtomography at 1.1μm voxel size with spatial resolution sufficient to visualize alveoli,^[^ ^42^ ^]^ though at substantially reduced contrast‐to‐noise ratio compared to equivalent post‐mortem scans. While lung imaging relies on contrast between soft tissue and air, brain imaging is more challenging, as the phase and the attenuation differences between soft tissues are much weaker. Additionally, the brain is surrounded by the skull, generally requiring a higher photon energy for imaging than ex vivo brains and thus providing less contrast in the soft tissue. The radiodense skull also introduces streak artifacts in reconstructions of the brain, as discussed by Croton et al. for in situ brain imaging of rabbit kittens.^[^ ^43^ ^]^ The total radiation dose that can be tolerated by living animals limits the spatial resolution that can be achieved at an acceptable contrast‐to‐noise ratio. Finally, there will be artifacts due to motion caused by breathing and cardiovascular action, even in anesthetized mice. The projection tiling approach used in this work is highly susceptible to such motion artifacts, as it is not robust to sample movements.

### 2.2 Registration to the Allen Mouse Brain Common Coordinate Framework

The microtmography volume was registered to the Allen Mouse Brain CCFv3 atlas. **Table** **1** lists the mean registration errors from twofold cross‐validation based on 70 manually selected landmark pairs. Results are shown for the configuration achieving the lowest errors for affine and non‐rigid registration as well as the best configuration when excluding image gradients (*w*_|∇**V**|_ = 0), segmentations (*w*_S_ = 0), and/or landmarks (*w*_L_ = 0). Lowest errors were achieved for affine and non‐rigid registration when landmarks were incorporated into the cost function, i.e., *w*_L_ > 0. The weights providing the lowest mean error for the twofold cross‐validation, shown in the last two columns in Table 1, were employed for the final registration. Note that all 70 selected landmarks were used for the final registration.

**Table 1:** Accuracy of image registration based on landmark error *f*_D_ from two‐fold cross‐validation are given for weight configurations of image gradient (*w*_|∇**V**|_), segmentation (*w*_S_), and landmarks (*w*_L_). For non‐rigid registration, meta‐parameters include bending energy penalty weight (*w*_BE_), which controls the smoothness of the deformation field, and control point grid spacing (*s*) in voxels. The lowest mean landmark error of 0.08 mm was achieved for non‐rigid registration with the configuration shown in the last column (gray). The other columns list the best configurations when excluding image gradients (*w*_|∇**V**|_ = 0), segmentations (*w*_S_ = 0), and/or landmarks (*w*_L_ = 0). For comparison, the mean landmark error after manual pre‐alignment was 2.49 mm.

	Affine registration						Non‐rigid registration					
*w*_|∇**V**|_	0	10^−8^	10^−8^	0	10^−8^	10^−8^	0	10^−8^	10^−8^	0	10^−8^	10^−8^
*w*_S_	0	0	1	0	0	1	0	0	1	0	0	1
*w*_L_	0	0	0	1	1	1	0	0	0	0.1	0.1	0.1
*w*_BE_	–	–	–	–	–	–	100	1000	100	1000	1000	1000
*s*	–	–	–	–	–	–	16	16	16	16	16	16
*f*_D_ [mm]	0.25	0.16	0.16	0.14	0.14	0.14	0.40	0.13	0.13	0.09	0.08	0.08

**Figure 2:**
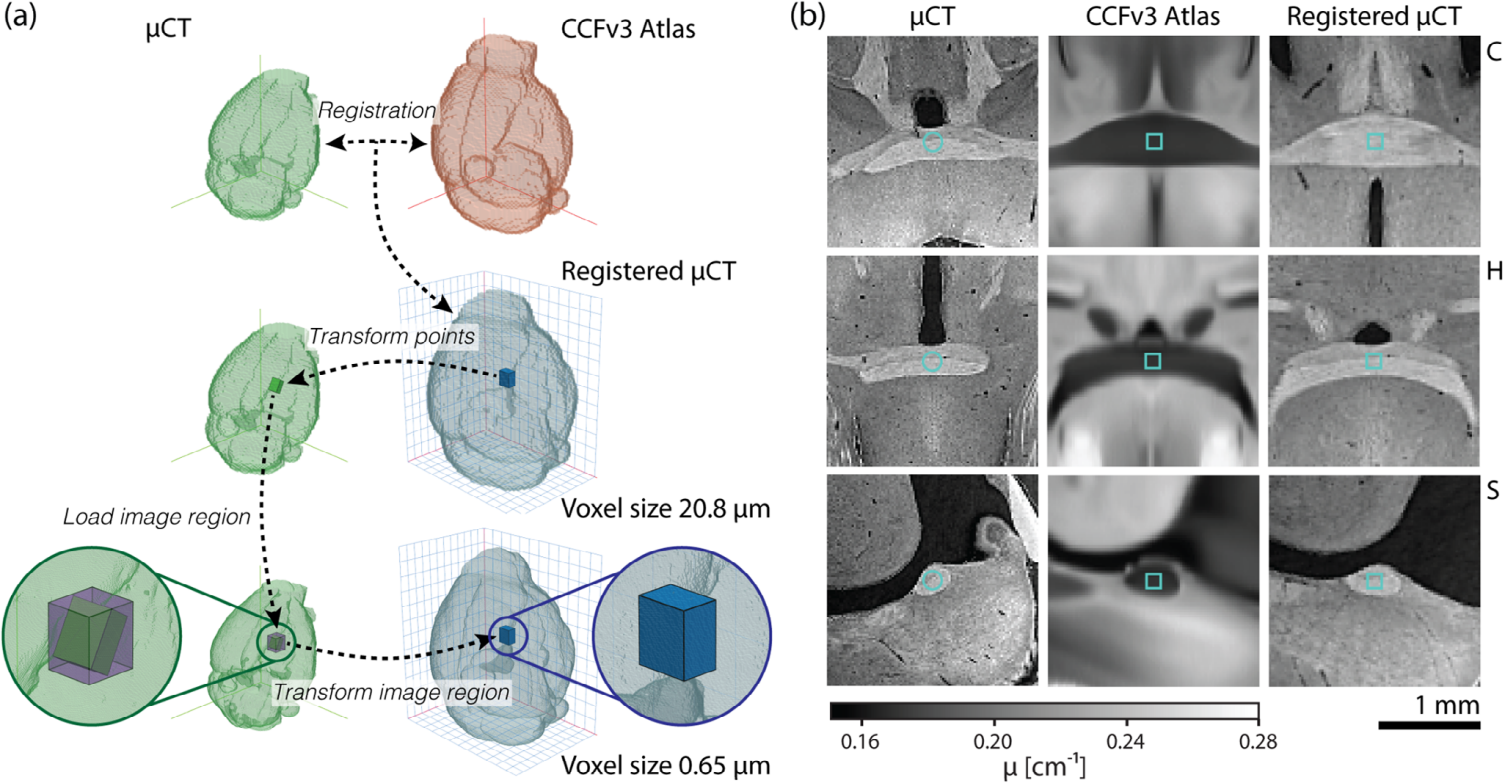
a) Illustration of the distributed hierarchical 3D image registration and transformation framework. (top) Registration was performed with the 32× downsampled microtomography (μCT) images to fit in available memory and have a resolution similar to that of the atlas. (middle) Given an axes‐aligned local image region in atlas space (blue box), the resulting spatial transformation provided the corresponding coordinates in the μCT image space (green box). (bottom) Local transformation of the original μCT image was then performed by loading only the axis‐aligned image region (purple box), which encloses the potentially skewed green box. Repeating this procedure for all 12 × 12 × 12 local regions in the reference space created the registered μCT image. b) Registration results at corresponding landmark positions in the original microtomography, the atlas, and the non‐rigidly registered μCT datasets are displayed in virtual coronal (C), horizontal (H), and sagittal (S) planes. Representative landmark pairs were selected by Observer 1 (cyan). Image intensities for the μCT and the two‐photon microscopy‐based template of the Allen Mouse Brain CCFv3 atlas were scaled to [1,99] percentile of pixel intensities. The color bar indicating the linear attenuation coefficient μ applies to the μCT images.

**Figure 3:**
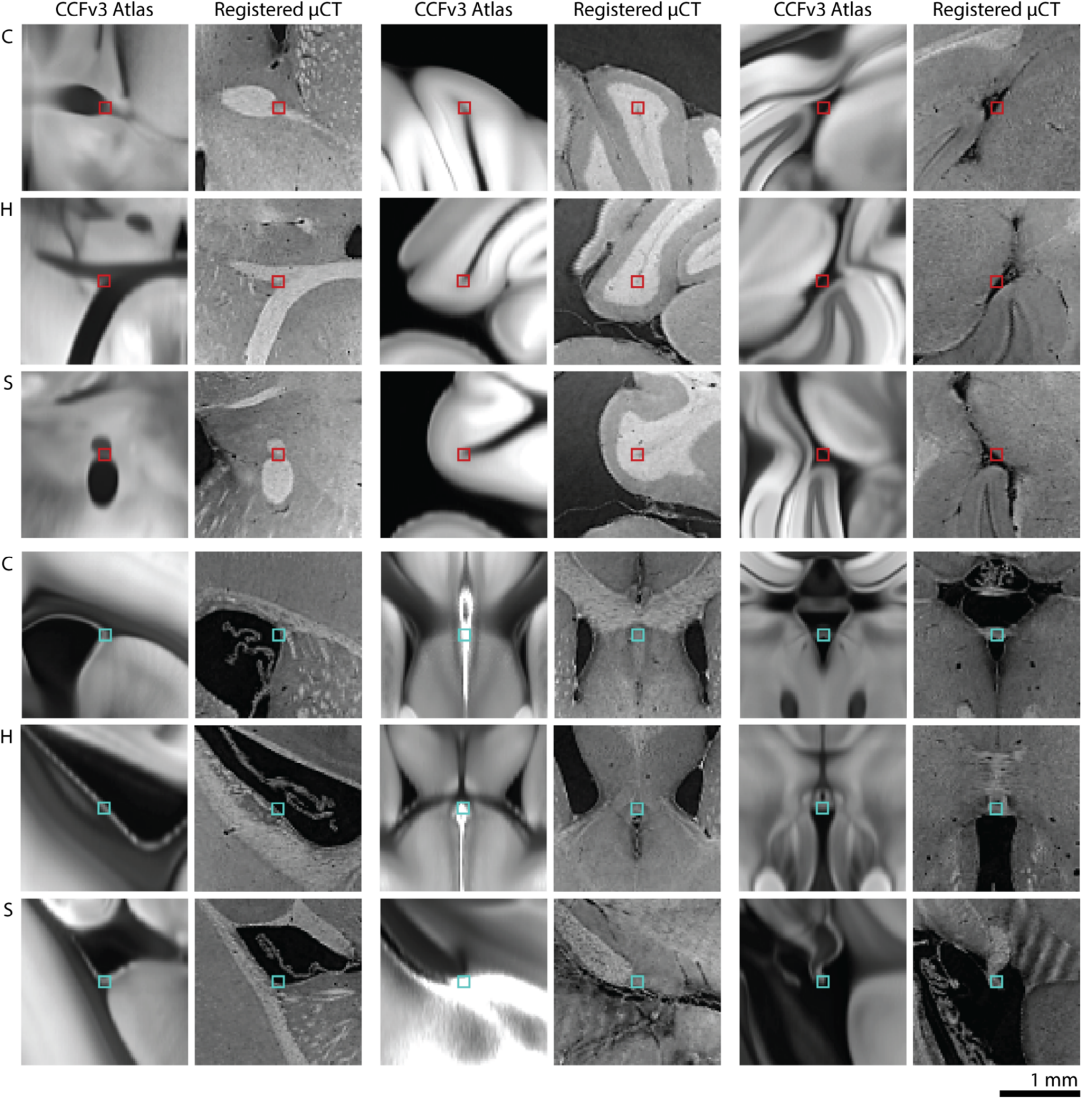
Registration results at corresponding landmark positions in the Allen Mouse Brain CCFv3 atlas and the non‐rigidly registered microtomography (μCT) datasets are displayed in virtual coronal (C), horizontal (H), and sagittal (S) planes. Representative landmark pairs selected by Observer 1 (bottom, cyan) and Observer 2 (top, red) are shown. Image intensities for the μCT and the two‐photon microscopy‐based template of the Allen Mouse Brain CCFv3 atlas were scaled to [1, 99] percentile of pixel intensities.

The number of confidently identifiable landmark pairs was limited by anatomical variations as well as differences in structure appearances between the microtomography volume and the average two‐photon microscopy CCFv3 template. Landmarks included ideal point landmarks as well as central positions of larger anatomical structures. **Figure** **2** (right) shows image regions around a manually selected landmark from Observer 1 in the original images and after registration. Visual inspection reveals that non‐rigid registration provided reasonable alignment of corresponding anatomical structures. The combination of intensity‐based registration and landmark alignment with optimized weighting supported robustness against small errors in manual landmark positions. Further landmarks and their alignment after registration can be seen in **Figure** **3**.

**Figure 4:**
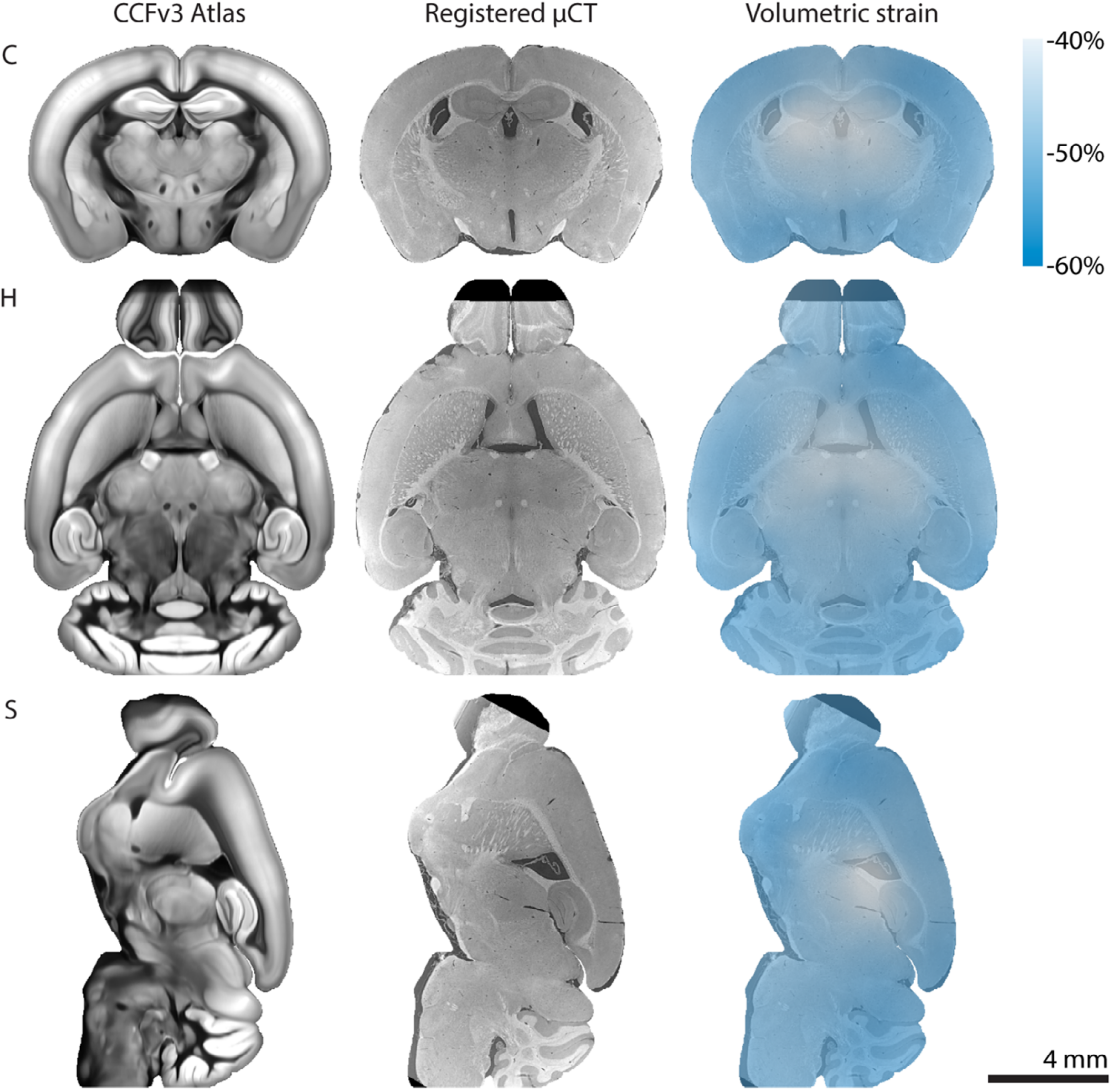
Non‐rigid registration results. Virtual coronal (C), horizontal (H), and sagittal (S) slices are shown from the two‐photon microscopy‐based template of the Allen Mouse Brain CCFv3 atlas,^[^ ^24^ ^]^ the non‐rigidly registered microtomography (μCT) volume, and the volumetric strain as a result of registration.

**Figure** **4** shows virtual slices from the atlas template image and the non‐rigidly registered microtomography volume. The resulting alignment enables the observation of similarities and differences in anatomy and appearance of structures. Due to the ill‐posed problem of registering multi‐modal data across mice, the alignment is imperfect in some regions. Yet, the alignment is sufficient to provide an understanding of the anatomical structures shown when overlaid with the atlas segmentation. Thus, the present registration is useful for general alignment with the Allen Mouse Brain CCFv3 atlas and navigation, but labels should not be transferred from the atlas without further refinement. Alignment with the CCFv3 also facilitates comparisons of the microtomography volume to histological slices from 132 coronal and 21 sagittal sections provided in the Allen Mouse Brain Reference Atlas.^[^ ^44^ ^]^

In the Allen Mouse Brain CCFv3 template the whole brain volume is 506  mm^3^ because the underlying serial two‐photon data of formalin‐fixed agarose‐immersed brains were scaled to match the unfixed fresh‐frozen Nissl‐based template of CCFv1.^[^ ^24^ ^]^ This achieved backward compatibility and closeness in volume to in vivo mouse brains.^[^ ^45^ ^]^ The mean brain volume in the original serial two‐photon data used for the CCFv3 template was 435  mm^3^.^[^ ^24^ ^]^ By comparison, transforming the CCFv3 template to the coordinate frame of our mouse brain dataset in 100 % ethanol produced a volume of 244  mm^3^. The local change in volume as a result of non‐rigid registration is shown in the right column of Figure 4. A smooth shrinkage pattern with a mean volumetric strain of −48 % can be observed, with larger shrinkage on the outside of the brain. We previously observed a median volumetric strain of −39 % from formalin to ethanol embedding,^[^ ^46^ ^]^ which here would correspond to an estimated volume of 400  mm^3^ in formalin and thereby falls within the range of volumes reported for the 1675 mouse brains used for the CCFv3 template.

### 2.3 Exploring and Disseminating the Full Mouse Brain Dataset

**Figure 5:**
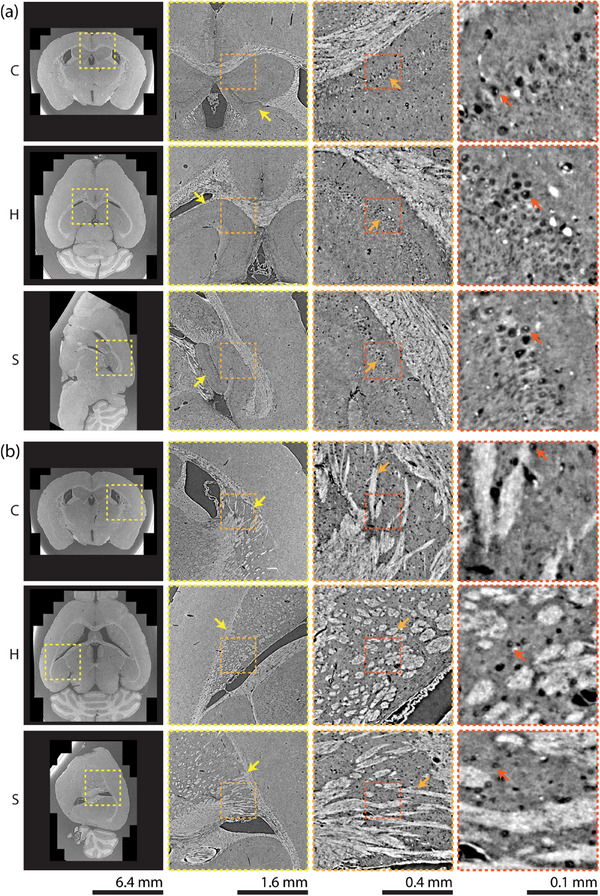
Microanatomical details from the a) hippocampal and b) striatum dorsal regions are shown in orthogonal virtual coronal (C), horizontal (H), and sagittal (S) slices of the microtomography volume registered to the Allen Mouse Brain CCFv3 atlas. a) Magnified views reveal structures of increasing detail, with arrows indicating (yellow) the hippocampus (including dentate gyrus and Ammon's horn), (orange) the CA1 field of Ammon's horn, and (red) the nucleus of an individual pyramidal neuron. b) Details from the striatum dorsal region are shown with arrows indicating (yellow) caudoputamen, (orange) fiber tracts, and (red) the nucleus of a single neuron. Multi‐resolution datasets are publicly shared in gzip‐compressed pre‐computed Neuroglancer format.

Details of microanatomical structures of the registered microtomography volume are shown in **Figure** **5** for the hippocampal and striatum dorsal region. The volumetric data can be virtually sliced along arbitrary planes. For example, Figure 5 shows six unique virtual orthogonal slices, while physical sectioning for standard histological analysis only allows for imaging of parallel planes along a predefined axis. At the highest resolution, cells, fibers, and other microscopic structures are clearly visible. The displayed virtual slices were downloaded from the publicly available dataset via CloudVolume, demonstrating that this dataset can not only be viewed, but also downloaded for processing.

**Figure 6:**
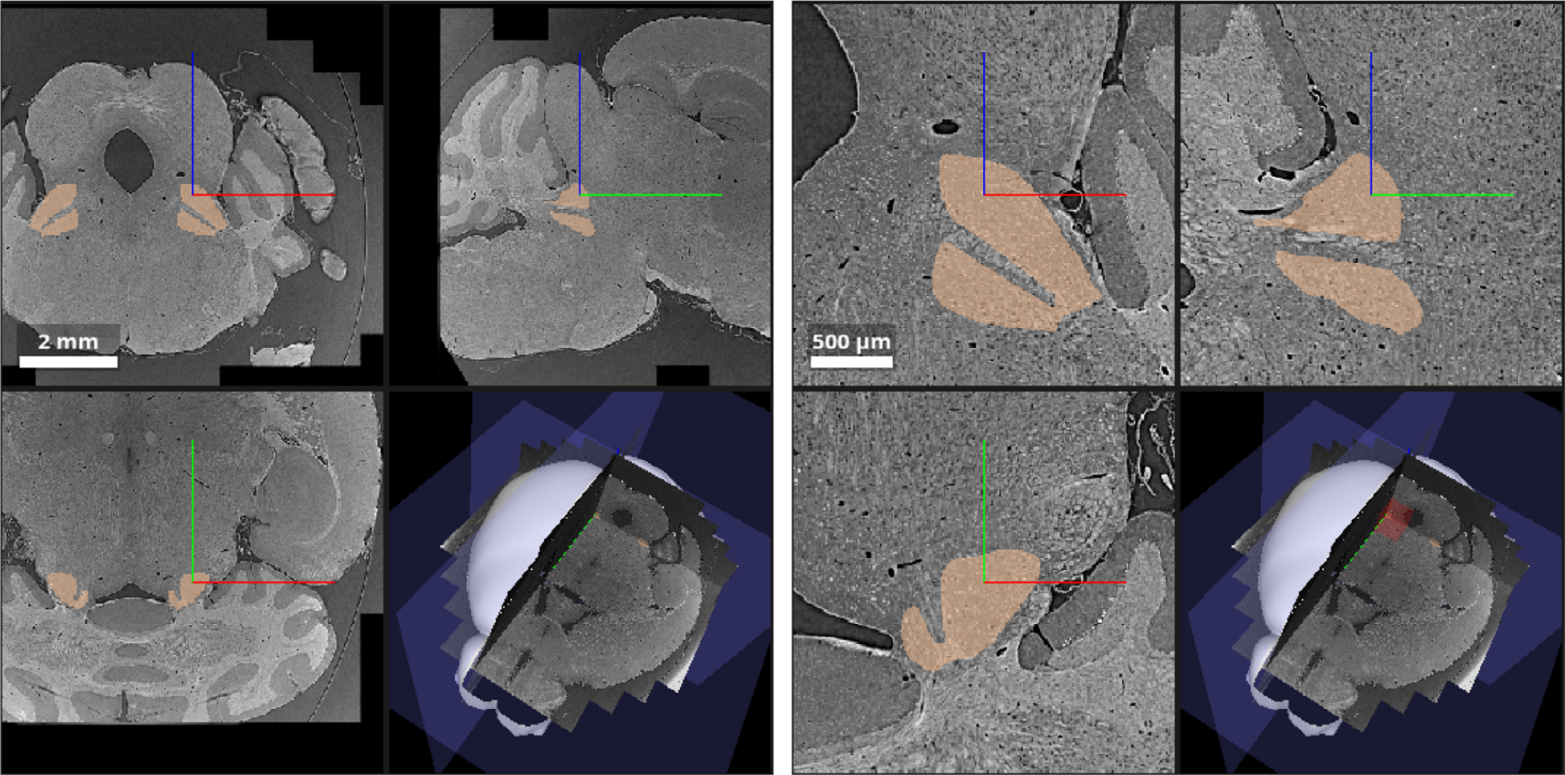
Browser‐based navigation of the publicly available mouse brain dataset. Virtual orthogonal slices from the microtomography data are displayed with an overlaid segmentation of the parabrachial nucleus region from the Allen Mouse Brain CCFv3 using the siibra‐explorer tool. The multi‐resolution viewer allows for exploring the full dataset at lower resolutions (left), while still loading the full‐resolution data at higher magnifications (right). The images are taken directly from screenshots of the siibra‐explorer displayed in a web browser. Scale bars in the upper left panels apply to all three orthogonal slices. Data are available at this URL.

Intensity values were stored as 16‐bit integers. These can be converted to linear attenuation coefficients by mapping the grayscale intensity range [0, 65535] to [−0.01 mm^−1^, 0.10 mm^−1^]. Note that negative attenuation values are the result of the distribution of air attenuation being centered at zero and phase‐contrast‐based edge enhancement that was not completely removed during phase retrieval. The gzip‐compressed pre‐computed data in Neuroglancer format required a total of 5.4 TB disk storage space. The complete multi‐resolution data have been uploaded to a publicly accessible ebrains repository^[^ ^47^ ^]^ and can be viewed via siibra‐explorer using this URL and in Neuroglancer using this URL. Note that both viewers allow encoding the position when sharing a URL, in this case located in the caudoputamen. Six resolution levels, in conjunction with the pre‐computed Neuroglancer format with data chunks of 64 × 64 × 64 voxels, allow fast interactive viewing via siibra‐explorer or Neuroglancer by loading only the required chunks at the appropriate image resolution. This eases navigation to microanatomical details and keeping the macroscopic overview. **Figure** **6** illustrates the visualization of the warped microtomography image in siibra‐explorer. The correspondence between the microtomography volume and the two‐photon template image of the Allen Mouse Brain CCFv3 can be observed. The microtomography volume can be explored by navigating to predefined brain regions of the Allen Mouse Brain CCFv3 such as the parabrachial nucleus.

## 3 Conclusion

Virtual histology based on extended‐field X‐ray microtomography provides isotropic micrometer resolution with sufficiently large FOV to image an entire mouse brain. The dataset presented here provides rich microanatomical detail and forms a basis for future analyses. Overcoming barriers in sharing, viewing, navigating, and combining these data with existing resources will unlock collaborations with domain experts who may have limited experience handling teravoxel‐sized imaging data. In this study, we introduced a dedicated data processing pipeline to manage terabyte‐sized virtual histology datasets and integrate them with open‐source interactive viewers. The reconstructed tomography data were registered and transformed into a common coordinate system, then made accessible through an online repository in a hierarchical format for exploration via web browser‐based viewers. Crucially, the data's compatibility with tools like siibra‐explorer and Neuroglancer allows users to apply existing labels from other datasets to rapidly identify areas of interest, overlay the microtomography volumes with images from other modalities, and share specific location coordinates via URL, facilitating collaboration with peers.

## 4 Experimental Section

### Preparation of the Mouse Brain

The imaged mouse brain came from a wild‐type 12‐week‐old female C57BL/6JRj mouse (Janvier Labs, Le Genest‐Saint‐Isle, France). Tissue collection was approved by the veterinary office of the Canton of Bern (license BE98/2020). The mouse was transcardially perfused with 4 % paraformaldehyde (PFA; Merck, Darmstadt, Germany) / phosphate buffered saline (PBS) pH 7.4 under Ketamine/Xylazine anesthesia, then the brain was dissected and immersed in 4 % PFA / PBS. For measurement, the brain was dehydrated in ethanol, which has been shown to enhance tissue contrast.^[^ ^46^, ^48^, ^49^, ^50^ ^]^ Dehydration was performed by 2‐h immersion in 20 mL of 50 %, 70 %, 80 %, 90 % and 100 % ethanol (Carl Roth GmbH, Karlsruhe, Germany).

### Extended‐Field Microtomography

The mouse brain was imaged at the anatomix beamline at Synchrotron soleil (Saint‐Aubin, France).^[^ ^17^, ^18^ ^]^ A polychromatic X‐ray beam (*white beam*) was used. The absence of reflective elements such as a crystal monochromator in the path of the filtered white X‐ray beam results in a particularly stable beam profile, reducing image artifacts. The energy spectrum of the beam was adapted to provide a reasonable trade‐off between contrast, which is typically better at lower photon energies, and intensity transmission through the object, and thus, signal‐to‐noise ratio in the images, which is typically better at higher photon energies. The selected configuration consisted of a succession of transmission filters with cumulative thicknesses of 600μm diamond, 26μm Au and 100μm Cu, resulting in an effective mean photon energy around 38keV. The undulator X‐ray source was operated at a magnetic gap of 5.5 mm, the minimum authorized value, to maximize the integral photon flux. Projections were recorded with an effective pixel size of 0.65μm on a scientific CMOS camera (Hamamatsu Orca Flash 4.0 V2, 2048 × 2048 pixels, 6.5μm physical pixel size) coupled to a 20‐μm‐thick LuAG scintillator via microscope optics with 10× magnification (numerical aperture 0.28, Mitutoyo Corporation, Kanagawa, Japan).^[^ ^51^ ^]^ An exposure time of 100 ms was selected to fill half of the detector's dynamic range, thus avoiding saturated pixels and reducing the severity of ring artifacts. As soft biological tissues have minute differences in X‐ray attenuation, the image contrast can be improved by considering the phase shift of the X‐rays. In propagation‐based phase contrast mode, refraction at material interfaces is made visible by allowing propagation of X‐rays between the object and the detector. Phase information can then be retrieved from radiographs through Fourier‐space filtering. Here, the detector was placed 50 mm downstream of the sample, which was close to the critical near‐field distance to ensure that propagation‐based blurring does not exceed two pixels.^[^ ^52^ ^]^

The detector's FOV was extended by a factor of eight both perpendicular to and along the axis of rotation, see Figure 1a–c. At each height, four 360°‐acquisitions were recorded with 9000 projections and offset rotation axis to allow for about 200 pixels of overlap. This scheme was repeated for eight height steps with about 200 pixels of overlap. Each individual acquisition took around 15 min using continuous rotation mode, resulting in a total scan time of about 8 h. The tilt of the rotation stage must be aligned to a precision better than 70μrad, as a reconstructed slice is only 0.65μm thick but 10 mm wide.

### Reconstruction of Extended Field‐of‐View Tomography Data

The processing of the extended‐field microtomography was divided into three steps, i) determination of the rotation axis and projection stitching positions, ii) blending of extended projections, phase retrieval, and ring artifact correction, iii) tomographic reconstruction. These steps were controlled by a parameter file and run as batch jobs, either locally or on scientific computing infrastructure such as sciCORE^[^ ^53^ ^]^ at the University of Basel. For the stitching of 8 × 8 images to one full projection per angle, see Figure 1b, the translational offsets between neighboring tiles were determined globally and then applied to each angle. The recorded motor positions were taken as an initial estimate for the translations between scans. The offset positions were further refined using sets of projections at ten equidistant angles by maximizing the normalized correlation coefficient in the overlapping region of adjacent frames. The final offset positions were taken as the mean of the values for these ten angles weighted by the peak prominence of the normalized correlation coefficient. Extended projections were assembled with these determined offset positions. For overlapping regions, the image intensities of adjacent tiles were combined with linear blending, where the original intensities *I*_1_ and *I*_2_ are mapped to a linear combination *I*_blend_ = α*I*_1_ + (1 − α)*I*_2_, with α ∈ [0, 1] denoting the position in the overlap region. Propagation‐based phase retrieval using the method introduced by Paganin et al.^[^ ^54^ ^]^ was applied, assuming a ratio of δ/β = 140 between the real and imaginary components of the refractive index *n* = 1 − δ + *i*β. This parameter was chosen based on visual inspection of trial reconstructions, striking a balance between contrast and spatial resolution.^[^ ^55^ ^]^ Note that the filter was modified to remove multiplication by the factor 1/μ (see Equation (3) in Weitkamp et al.^[^ ^52^ ^]^). This enables the interpretation of reconstructed values as linear attenuation coefficient. For ring artifact correction, the mean of flat‐field‐corrected projections was taken over all rotation angles. A high pass filter was applied to isolate inhomogeneities leading to ring artifacts. This filtered mean projection was subtracted from all projections. The flat‐field corrected, phase‐retrieved, and blended projections were stored in Tagged Image File Format (TIFF) with tiling in 32‐line strips for quick block‐wise reading.^[^ ^56^ ^]^ Overall, 4495 projections were produced, each measuring 14982 × 14982 pixels. Tomographic reconstruction was performed using a Fourier space re‐gridding reconstruction algorithm^[^ ^57^, ^58^ ^]^ implemented in tomopy (version 1.4.2)^[^ ^59^ ^]^ on blocks of 32 sinograms. The reconstructed slices were rescaled to 16‐bit signed integer range representing attenuation values spanning [−0.01 mm^−1^, 0.10 mm^−1^] and stored in TIFF format.

The spatial resolution of the reconstructed data was estimated using the Fourier shell correlation (FSC).^[^ ^60^ ^]^ A 154 × 1020 × 1020 voxel volume located in the overlap of height steps three and four was reconstructed from two independent sets of projections. The two resulting volumes were multiplied with a Hamming window^[^ ^61^ ^]^ to remove discontinuities at the borders.^[^ ^62^, ^63^ ^]^ The FSC curve was smoothed by applying a third order Savitzky–Golay filter^[^ ^64^ ^]^ with window length 50. The spatial resolution, defined as the width of the smallest resolvable line pair, was determined as the inverse of the crossover frequency of the smoothed FSC with the 3σ‐curve.^[^ ^65^ ^]^ This resulted in a spatial resolution of 1.7μm (full modulation period).

Volume renderings of the reconstructed data were prepared with VGStudio MAX 2.1 (Volume Graphics, Heidelberg, Germany) and are shown in Figure 1.

### Registration to the Allen Mouse Brain Common Coordinate Framework

Three‐dimensional registration of large volumes requires substantial computational resources. For example, in a prior study the full mouse brain volumes had to be downsampled to 9.3‐μm‐wide voxels and the number of transformation parameters had to be limited to one displacement vector per 12^3^ voxels to fit into the 144 GB memory of the used workstation.^[^ ^46^, ^66^ ^]^

To address this challenge, a distributed hierarchical method involving sub‐volume registration was developed for the present full‐resolution data. The process is illustrated in Figure 2. Initially, the volumes were downsampled to a size that allows them and the associated transformation parameters to be stored in memory during the image registration process. Subsequently, image registration was conducted for these low‐resolution volumes. The resulting spatial transformation was employed to define corresponding local regions (Figure 2, left, middle row). Axes‐aligned regions of interest in the full‐resolution volumes can then be processed independently. This involves transforming them in accordance with the low‐resolution registration outcomes, as illustrated in Figure 2 (bottom row), and/or registering them in instances where the degree of downsampling prevented accurate alignment of fine anatomical structures.

This pipeline was employed for registering the microtomography volume to the template image of the Allen Mouse Brain CCFv3,^[^ ^24^ ^]^ namely a hemisphere‐symmetric population average volume generated from two‐photon microscopy images of 1675 mouse brains. For image registration, we used the open‐source software elastix ^[^ ^67^, ^68^ ^]^ (version 5.0), as it offers standard registration functionalities as well as further useful features such as incorporation of landmark correspondences in the optimization function and definition of image regions to be registered.

The tomography data were downsampled by a factor of 32 × 32 × 32, i.e., to isotropic voxels of 20.8μm size, to approximate the resolution of the atlas, which has an isotropic voxel size of 25.0μm. Volumes were first manually aligned via a similarity transformation using ITK‐SNAP ^[^ ^69^ ^]^ (version 3.8.0). To tune and guide registration, two observers each manually selected 35 corresponding landmark pairs **L**_F_ and **L**_M_, where subscripts F and M denote fixed and moving image space, respectively. These landmarks from both observers were split into two equally sized sets to perform two‐fold cross‐validation to determine suitable registration meta‐parameters. Besides the image volumes, i.e., the fixed atlas template **V**_F_ and the moving microtomography **V**_M_, image gradient magnitudes |∇**V**_F_| and |∇**V**_M_| as well as full brain segmentations **S**_F_ and **S**_M_ were used in a multi‐image, multi‐metric registration. Thus, the spatial transformation *T* was determined by minimizing the cost function *C* given in Equation (1):

(1) $$\begin{array}{c} \begin{array}{cc} C & =f_{-\mathrm{MI}}\left(I_{F},T\left(I_{M}\right)\right)+w_{\left|\nabla V\right|}f_{\mathrm{MSD}}(|\nabla V_{F}|,T(|\nabla V_{M}\left|)\right)\\ & \;+w_{S}f_{-\mathrm{KS}}\left(S_{F},T\left(S_{M}\right)\right)+w_{L}f_{D}\left(T\left(L_{F}\right),L_{M}\right)+w_{\mathrm{BE}}f_{\mathrm{BE}}\left(T\right) \end{array} \end{array}$$

The cost function *C* includes terms for i) negative mutual information *f*_−MI_ to account for the non‐linear relationship between the two modalities, ii) mean squared difference *f*_MSD_ of the image gradient magnitudes to ensure edge alignment, iii) negative Kappa statistics *f*_−KS_ to maximize overlap between segmentations, iv) mean Euclidean distance *f*_D_ between landmarks to guide alignment, and v) bending energy penalty *f*_BE_ to promote smooth transformations. To include the brain boundaries, image dissimilarity measures *f*_−MI_, *f*_MSD_ and *f*_−KS_ were determined for an extended brain region, i.e., within **S**_F_ and **S**_M_ dilated by a sphere of radius ten voxels. We tested the usefulness of incorporating |∇**V**| and **S** in the registration, since the image gradient magnitudes will guide the local alignment of edges and the segmentations will support global alignment. The bending energy is defined as the sum of the second‐order spatial derivatives of the transformation,^[^ ^70^ ^]^ and the related cost term *f*_BE_ penalizes sharp changes in the non‐rigid transformation.

Registration was based on manual alignment with a similarity transformation, followed by automatic affine and then deformable registration using a grid of control points with a spacing of *s* voxels interpolated with cubic B‐Splines. Based on initial tests and the relative values of the cost functions, the registration meta‐parameters were tested in the following ranges: *w*_|∇**V**|_ ∈ {0, 10^−8^}, *w*_S_ ∈ {0, 1}, *w*_L_ ∈ {0, 0.1, 1}, *w*_BE_ ∈ {1, 10, 100, 1000, 10000, 100000}, and *s* ∈ {32, 16}. Finally, the images were registered using 70 landmarks and registration meta‐parameters providing the lowest mean error for both cross‐validation folds.

The full resolution tomography volume (moving volume) was warped to the atlas (fixed volume) by dividing the fixed space into 12 × 12 × 12 target subregions, such that the axes‐aligned extended moving image subregion (purple bounding box in bottom row of Figure 2), the moving image transformed subregion (blue box in bottom row of Figure 2), and the transformation parameters all fit in memory for an isotropic target voxel resolution of 0.65μm.

### Visualization and Sharing of TB‐Sized Data

For dissemination and exploration of the registered data, siibra‐explorer and Neuroglancer were used. Neuroglancer is an open‐source browser‐based interactive visualization platform that supports datasets up to petabyte size.^[^ ^26^ ^]^ The warped microtomography volume was transformed into gzip‐compressed chunks of 64 × 64 × 64 voxels in sharded pre‐computed Neuroglancer format at six resolution levels, i.e., 0.65, 1.3, 2.6, 5.2, 10.4 and 20.8μm for efficient visualization. The sharded format combines all chunks into a fixed number of larger shard files to avoid the performance penalties incurred by many small files. For this dataset, the number of required files was substantially reduced from 20312064 to 4959. The sharded chunks were produced using the open‐source software Igneous (version 4.19.2).^[^ ^71^ ^]^ The pre‐computed data were uploaded to an ebrains repository, where they can be publicly accessed with siibra‐explorer or the Neuroglancer viewer using the corresponding URL. In siibra‐explorer, users can navigate to more than 1300 predefined Allen Mouse Brain CCFv3 regions by their anatomical names using the underlying hierarchical ontology.^[^ ^24^ ^]^ The data can also be accessed and processed by suitable software including CloudVolume ^[^ ^72^ ^]^ in combination with Igneous.

## Conflict of Interest

The authors declare no conflict of interest.

## Data Availability

The data that support the findings of this study are openly available in EBRAINS at https://doi.org/10.25493/JWM7‐2G1, reference number 1, and in the FABRIC4 data portal at https://doi.org/10.5281/zenodo.11234384.
